# AP-1 Transcription Factors as Regulators of Immune Responses in Cancer

**DOI:** 10.3390/cancers11071037

**Published:** 2019-07-23

**Authors:** Vasileios Atsaves, Vasiliki Leventaki, George Z. Rassidakis, Francois X. Claret

**Affiliations:** 1 Department of Oncology, Ludwig Institute for Cancer Research-Lausanne Branch, University of Lausanne, Épalinges, 1066 Lausanne, Switzerland; 2 Department of Pathology, Children’s Hospital of Wisconsin & Medical College of Wisconsin, Medical College of Winsconsin, Milwaukee, WI 53226, USA; 3 Department of Oncology-Pathology, Karolinska Institutet, Karolinska University Hospital, 17176 Stockholm, Sweden; 4 Department of Pathology and Cytology, Karolinska University Hospital, 17176 Stockholm, Sweden; 5 Department of Systems Biology, The University of Texas-MD Anderson Cancer Center, Houston, TX 77030, USA

**Keywords:** AP-1, immune checkpoints, PD-1, CTLA-4, PD-L1, immunotherapy, targeted therapy, transcription factors, Tregs

## Abstract

Immune check point blockade therapy has revolutionized the standard of cancer treatment and is credited with producing remarkable tumor remissions and increase in overall survival. This unprecedented clinical success however is feasible for a limited number of cancer patients due to resistance occurring before or during a course of immunotherapy, which is often associated with activation of oncogenic signaling pathways, co-inhibitory checkpoints upregulation or expansion of immunosuppressive regulatory T-cells (Tregs) in the tumor microenviroment (TME). Targeted therapy aiming to inactivate a signaling pathway such as the Mitogen Activated Protein Kinases (MAPKs) has recently received a lot of attention due to emerging data from preclinical studies indicating synergy with immune checkpoint blockade therapy. The dimeric transcription factor complex Activator Protein-1 (AP-1) is a group of proteins involved in a wide array of cell processes and a critical regulator of nuclear gene expression during T-cell activation. It is also one of the downstream targets of the MAPK signaling cascade. In this review, we will attempt to unravel the roles of AP-1 in the regulation of anti-tumor immune responses, with a focus on the regulation of immune checkpoints and Tregs, seeking to extract useful insights for more efficacious immunotherapy.

## 1. Introduction

The 2018 Nobel Prize for Physiology or Medicine awarded to the pioneers of immune checkpoint research, James P. Allison and Tasuko Honjo, attests to the considerable advances achieved in the field of immunotherapy over the past decades. Immunotherapy, and more specifically immune checkpoint blockade therapy, represents a transforming event in the treatment of metastatic cancer since for the first time studies have shown that it can promote strong and durable tumor regression in some patients whose tumors have already metastasized [1].

Immune checkpoint blockade (ICB) or immune check point inhibition therapy aims at the inhibition of the molecules collectively known as immune checkpoints, expressed mainly on the cells of the immune system. Immune checkpoints, whose physiological role is to maintain self-tolerance and restrict collateral damage in the tissues following immune responses, are frequently exploited by tumor cells to escape from the surveillance of the immune system which leads to immune suppression and promotion of tumor growth [2]. In comparison to conventional treatment modalities in oncology (radiotherapy, chemotherapy etc.), ICB is innovative because it targets molecules expressed on the cells of the immune system, aiming to disrupt the tumor-derived immune suppression and reinvigorate the immune system to elicit a potent and oftentimes durable anti-tumor response. Monoclonal antibodies targeting immune checkpoints (e.g., CTLA-4 and PD-1) have so far received approval from the Food and Drug Administration (and other regulatory authorities) for demonstrating dramatic clinical responses in patients with metastatic cancers, leading to substantial improvement of their overall survival (OS) across diverse histological types and genetics of neoplastic diseases [2,3]. 

Emerging evidence, however, suggests that ICB is beneficial only for a fraction of cancer patients, while it comes with a great cost of severe immune-related adverse events (irAEs) for a significant portion of those treated [4]. Data from several clinical trials suggest that patients are stratified in the following three groups according to their resistance to ICB therapy: (1) responders, patients who respond to initial treatment and continue to respond, (2) non-responders, those that do not succeed in responding to ICB (primary resistance), and (3) those that initially respond but eventually develop disease progression (acquired or adaptive resistance) [5,6]. Several factors have been identified within the TME, such as a genetic and epigenetic mutational load that controls neoantigen processing or presentation by the tumor cells, inhibitory checkpoint expression (e.g., PD-L1) and activation of oncogenic signaling pathways (tumor cell intrinsic mechanism) that suppress the therapeutic effects of ICB by disrupting the functions of tumor-specific cytotoxic T cells [2,7]. In addition, tumor cell extrinsic mechanisms operating outside the TME such indole 2,3-dioxygenase (IDO) activity and the actions of immunosuppressive cells like T regulatory (Tregs) and myeloid-derived suppressor cells (MDSCs), is conducive towards an immunosuppressive environment, promoting tumor growth and resistance to ICB [7].

A growing body of evidence indicate that activation of signaling pathways in various cancer types, such as the Mitogen-Activated Protein Kinases (MAPK) [8], phosphatidylinositol 3-kinase (PI3K) [9,10] and wnt/β-catenin [11] can promote an immune-compromised tumor microenvironment, conferring resistance to immunotherapy. Preclinical evidence in various cancer types suggest that MAPK inhibition (MAPKi) can dramatically increase the efficacy of immunotherapy [12,13,14,15,16,17] mainly via increased antigen presentation from tumor cells, augmented MHC-I expression, suppressed Treg expansion and increased proliferation and activation of tumor-infiltrating cytotoxic T-cells.

Currently, several combinatorial treatment strategies with ICB and MAPKi are under investigation (Table 1, summary of clinical trials) and early results from these clinical trials indicate synergistic inhibition of tumor growth and beneficial effect for patients [18]. For instance, a phase II trial of dabrafenib, trametinib, and nivolumab in BRAF-mutated advanced melanoma patients showed an overall response rate 91% with relatively small proportion of patients that discontinued the study due to drug toxicity [19]. In a phase III trial (COMBI-I), which evaluated dabrafenib, trametinib and PDR001, an anti-PD-1 antibody, in patients with advanced BRAF-mutated melanoma, preliminary results show that all nine patients responded (33% complete responses and 67% partial responses) [20]. Therefore, the initial response rates for combination of targeted therapies with immunotherapy show promising results despite the reported toxicities. Although these studies are promising, the exact players linked to the MAPK inactivation that are responsible for modifying the TME and make it amenable to immunotherapy remain largely elusive. 

**Figure 1:**
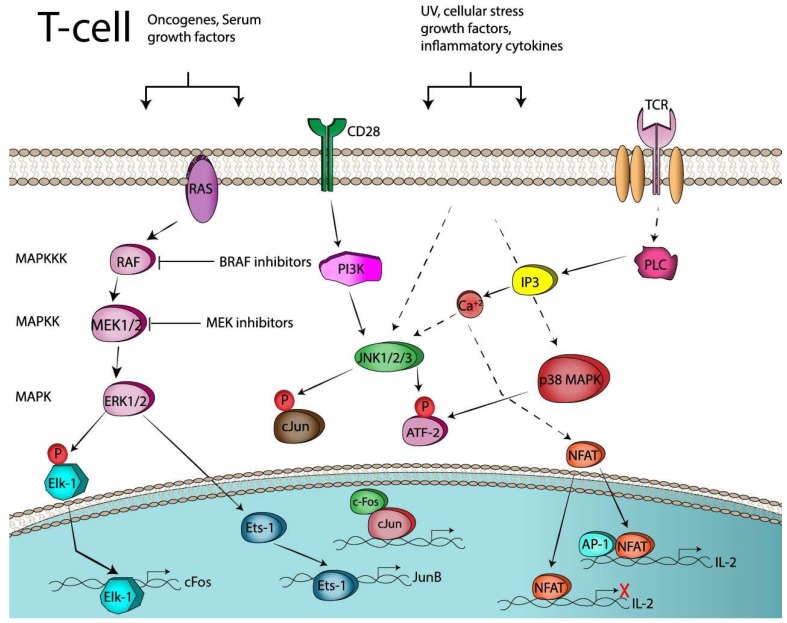
Transcriptional and post-translational activation of AP-1 in T-cells. UV (Ultraviolet); CD28 (co-stimulation, signal-2), TCR (T-cell receptor, signal-1).

The Activator protein-1 (AP-1), is a group of transcription factors consisted of four sub-families: the Jun (c-Jun, JunB, JunD), Fos (c-Fos, FosB, Fra1, Fra2), Maf (musculoaponeurotic fibrosarcoma) (c-Maf, MafB, MafA. Mafg/f/k, Nrl), and the ATF-activating transcription factor (ATF2, LRF1/ATF3, BATF, JDP1, JDP2) protein families [21], characterized by pleiotropic effects and a central role in different aspects of the immune system such as T-cell activation, Th differentiation, T-cell anergy and exhaustion [22,23]. MAPK signaling cascade is of paramount importance [24] for regulating AP-1 transcriptional activation and DNA binding activity on a wide array of AP-1 target genes (Figure 1). In the present review, we will be focusing on the specific biologic impact of AP-1 transcription factors on the regulation of immune checkpoints and function of Tregs, both of which can be contributing factors in resistance to ICB. Therefore, a comprehensive description of other important oncogenic functions of AP-1 transcription factors is out of the scope of this article.

AP-1 activity is controlled by the Mitogen Activated Protein Kinases (MAPK), a family of enzymes conserved among eukaryotes that regulate cellular activities in response to numerous environmental signals (e.g., oncogenes, cytokines, growth factors). These signals result in activation of the MAPK pathway via a cascade of phosphorylation events on serine/threonine residues of distinct target proteins, peaking in the activation of the extracellular signal-regulated kinase (ERK), the c-Jun N-terminal kinase (JNK) and the p38 kinase. Inactivation of the pathway is possible by specific pharmacological inhibition of upstream signaling nodes, MAPKKK (MAPK Kinase Kinase) and MAPKK (MAPK Kinase). ERKs (ERK1/2), activate by phosphorylation Elk-1 a transcription factor that belongs to a family classified as a ternary complex factor (TCF). Elk-1 binds the promoter of *c-FOS* and rapidly induces its expression, contributing to the formation of the transcriptionally active dimers between Fos:Jun, which exhibit high transactivation potential to regulate a wide array of AP-1 target genes. ERKs can also activate JunB transcription by activating Ets-1, an ETS-domain transcription factor that augments the expression of Fos and Jun family members (e.g., JunB) through direct binding on the respective gene promoter. The JNKs, phosphorylate cJun at the transactivation domain (ser63, ser73) and ATF-2 within its N-terminal activation domain (Thr63, Thr71) and thus potentiate the transactivation capacity of these AP-1 members. ATF-2, was also found to be a substrate for p38 kinase, the third member of the MAPK, through phosphorylation at Thr69 and Thr71, which have important implications for its activation. During T-cell activation, TCR/CD28 signaling via PI3K and PLC (generation of Ca^+2^ through IP3) converge to the JNK activation which, in turn, leads to increased AP-1 activity. These transcriptionally active AP-1 components, form cooperative hetero-dimers with the NFAT transcription factor and control the transactivation of key molecules involved in T-cell responses like the IL-2 gene by binding to composite DNA elements. Finally, lack of AP-1 proteins signify that “partnerless” NFAT will bind the target genes with low transactivation potential leading to cell exhaustion or anergy. AP-1 members: cJun, JunB, c-Fos and ATF-2.

## 2. Activator Protein-1 (AP-1) Transcription Factors

It has been more than 30 years since the discovery of activator protein-1 (AP-1), described initially as a DNA-binding protein which recognized a DNA element found in the enhancer region of SV40 and the human Metallothionein IIA gene (*MT2A*) [25] and characterized by their ability to transactivate target genes upon phorbol ester stimulation (TPA) [26]. These AP-1 transcription factors were later found to regulate a wide range of cellular processes spanning from cell proliferation and survival to tumor transformation, differentiation and apoptosis [21]. AP-1 transcription factors are homo- or hetero-dimmer forming proteins that belong to a group of DNA binding proteins called Basic -Leucine Zipper domain (bZIP) [27]. Dimerization between members of the AP-1 family occurs through a structure which is known as leucine zipper, comprised of a heptad of repeats of leucine residues along a α-helix, which can dimerize with another α-helix via formation of a coiled–coil structure with contacts between hydrophobic leucine zipper domain. Adjacent to the leucine zipper lies a basic DNA binding domain which is rich in basic amino acids and is responsible for DNA-binding in either 12-*O*-tetradecanoylphorbol-13-acetate (TPA) response elements (5′-TGAG/CTCA-3′) or cAMP response elements (CRE, 5′-TGACGTCA-3′) [21,28]. Members of the AP-1 protein family differ markedly in their potential to transactivate AP-1 responsive genes and their ability to form dimmers. For example, the Fos sub-family cannot homodimerize, but they can form stable heterodimers with Jun members [29,30]. The Fos and Jun proteins have high transactivation potential, whereas others like JunB, JunD, Fra-1 and Fra-2 are weaker [31]. Early studies using murine fibroblasts, substantiate the antagonistic nature of some AP-1 members against others. For instance, cJun transcriptional activity is attenuated by JunB and this is due to differences in their activation domains [31,32]. Nevertheless, the current viewpoint suggests that the differential expression of AP-1 components and the cell context of their interactions determines the complex functions of AP-1 transcription factor [27]. 

### 2.1. Regulation of Immune Response by AP-1 in Genetically Modified Mice

Early studies from transgenic animals implied a prominent role of AP-1 in the regulation of the immune system, arising from targeted overexpression of JunB in T lymphocytes [33]. In these transgenic mice, elevated JunB levels caused upregulation of IL-4 expression, a cytokine which is known for facilitating the differentiation of Th2 cells while preventing the differentiation of Th1 cells. JunB binds to P1 element on *IL-4* promoter and synergizes with c-Maf to activate IL-4 luciferase reporter gene and JunB is also preferentially upregulated in developing Th2 cells. Collectively, this study suggests that JunB may contribute to the differentiation of naïve T-helper cells into Th2 during T cell development. In addition, in vivo data from transgenic mice expressing a mutant variant of cJun (JunAA), which is unable to sustain activation by JNK phosphorylation, reveal that even though T-cell activation and proliferation were not impaired in these mice, c-Jun N-terminal phosphorylation was required for efficient TCR- and TNFa (tumor necrosis factor-α)-induced thymocyte apoptosis, suggesting a role for cJun in thymocyte development [34]. On the other hand, ectopic expression of the FosB2 gene in thymocytes causes aberrant development of T cells and thymic epithelial cells [35]. 

### 2.2. AP-1 and T-Cell Activation

T-cell activation of naïve T-cells requires two signaling events [36]. The initial signal, signal-1, is generated by interaction of a peptide antigen presented in association with an MHC molecule on the surface of an antigen presenting cell (APC). The supply of a subsequent co-stimulatory second signal (signal-2) which is delivered by interactions of CD28 on the T-cell with molecules on the APC is then required for full T-cell activation and production of cytokines (IL-2), proliferation and differentiation of effector cells [37]. The signaling pathways that are activated by both signals (signal 1 and signal 2) are now well identified [38] and they culminate in the activation of the enzyme phospholipase C (PLCγ), which cleaves the membrane lipid PI(4,5)P2 (phosphatidylinositol 4,5 bisphosphate) producing the second messengers IP3 and DAG (diacylglycerol). The first message, IP3, results in a rapid release of Ca^2+^ from the ER and this will eventually lead to the activation of the transcription factor, nuclear factor of activated T-cells (NFAT) (Figure 1). Ras, a small G protein which is dependent on GTP for activation, will initiate following TCR engagement the signaling cascade of MAPK which leads to phosphorylation of ERKs (Erk1/2) and the activation of Elk1 by phosphorylation. The latter translocates to the nucleus and binds to the promoter of *C-FOS*, thus facilitating its transcription (Figure 1) [39]. An additional mechanism contributing to AP-1 activation from TCR engagement stems again from the MAPKs and the activation of the Jun-N-terminal kinase (JNKs) [40] (Figure 1). The JNKs, phosphorylate cJun, enhancing its transcriptional activity, leading to the formation of the AP-1 complex, Jun:Fos heterodimers, which are transcriptionally potent and thus bind and regulate target genes. cJun in several cancers (melanoma, colon cancer) is also induced through a MAPK independent mechanism, which involves cell–cell contacts and the adhesion molecule E-cadherin [41]. Also, the CD28 pathways via PI3 kinase and acidic sphingomyelinase, can lead to the induction of AP-1 [42]. The effects of the AP-1 transcription factors, associated with the immune system, are largely mediated through combinatorial regulation with the NFAT, a calcium/calcineurin pathway-dependent transcription factor. The transcription factors of the NFAT family are key regulators of T cell activation [43]. There are five members in the NFAT family members, of which NFAT1-4 (NFATc1-c4) are regulated by Ca^2+^-calcineurin signaling. NFAT/AP-1 transcription factors bind cooperatively to composite DNA sites, where they participate in the formation of stable ternary complexes regulating the expression of target genes (Figure 1). Composite DNA sites have been identified on the promoters of most of the cytokine genes, including *IL-2, IFN-g, TNF-a, GM-CSF, IL-4, FasL, CD25* and *NFAT/AP-1* where combinatorial regulation has been well documented [44]. DNA-binding experiments have demonstrated that the NFAT/AP-1-binding complex contains predominantly cJun, c-Fos, JunB and Fra-1 proteins [45]. 

### 2.3. AP-1 and T-Cell Anergy or Exhaustion

T-cell “anergy” is an unresponsive state of T-cells in which T-cells are activated in the absence of a positive costimulatory signal, while T-cell “exhaustion” is referred to the state of CD8^+^T cells that respond poorly because of prolonged antigen exposure during chronic viral infections or cancer [46,47]. Some of the hallmarks of anergic T cells are the inhibition of proliferation and their inability to synthesize IL-2 in response to TCR engagement [46]. Similarly, exhausted CD8^+^T cells display a transcriptional program distinct from that of functional effector or memory CD8^+^ T cells, characterized by the expression of inhibitory cell-surface receptors, including PD-1, LAG-3, TIM-3, TIGIT, and CTLA-4, and by impaired IL-2, TNF, and IFN-γ cytokine production. NFAT and AP-1 transcription factors synergistically play a central role in inducing hyporesponsive states, such as anergy and exhaustion [48,49]. Exhausted cells exhibit low expression of AP-1 factors (Fos, Fosb, and Junb) [50]. In addition, several lines of evidence suggest that cooperation of NFAT with AP-1 stimulates gene expression after immune response while absence of AP-1 leads to repression of the involved genes and to blockade of T-cell activation and proliferation, which eventually leads to T-cell anergy [51,52]. NFAT, in the absence of cooperation with the transcription factor AP-1 (Fos-Jun), fosters a transcriptional program of genes associated with anergy and exhaustion in both CD4^+^ and CD8 T cells, whereas, when AP-1 factors are present, NFAT drives expression of molecules such as cytokines that are involved in effector responses [48]. 

Collectively, these findings indicate that the presence or absence of AP-1 in the transcriptional complexes with NFAT contributes greatly to the anergy/exhaustion phenotype of T-cells, reversal of which is an important clinical goal as demonstrated by the immune checkpoint blockade therapy. Therefore, targeting the AP-1:NFAT complexes might have therapeutic implications. Indeed in a recent study, Mognol et al. designed a FRET-based high-throughput screen to identify compounds that disrupt the NFAT:AP-1:DNA complex. They identified a small molecule, which disrupts the NFAT:AP-1 interaction at the composite antigen-receptor response element-2 site without affecting the binding of NFAT or AP-1 alone to DNA. This small molecule is capable of binding to DNA in a sequence-selective manner and inhibit the transcription of the IL2 gene and several other cyclosporin A-sensitive cytokine genes important for the effector immune response thus providing a proof-of-concept approach to target AP-1 transcription factors [53].

## 3. AP-1 and Immune Checkpoint Regulation

According to the two-signal model, co-stimulatory molecules are responsible for sustained T-cell activation and effector T-cell function. Evidence for the two-signal model of T-cell activation was provided by the discovery of CD28 on T-cells, as the archetypal co-stimulatory molecule, which after binding to its ligand, it provides signal-2 stimulation, which along with TCR signal-1 is required for full T-cell activation. Subsequent discovery of CTLA-4 as an antagonistic molecule to the CD28 function, provided feedback for the negative stimulation of T-cells following activation and designated a group of molecules with similar function as co-inhibitory molecules. Ever since, the list of co-stimulatory and co-inhibitory molecules and their ligands is exponentially increasing along with potential clinical applications in patients with more than 10 cancer types, including metastatic melanoma, renal cell carcinoma, non-small-cell lung carcinoma (NSCLC), Hodgkin’s lymphoma and several others [54,55,56]. Signaling downstream the immune checkpoint molecules is complex and has been reviewed in great extent elsewhere [57], but there is evidence for the participation of several members of the AP-1 family [57].

### 3.1. Co-Stimulatory Molecules and AP-1 Transcription Factors

#### 3.1.1. CD28

CD28, a 44-kDa type I transmembrane glycoprotein, is constitutively present on the surface of naïve and activated T-cells [58]. Stimulation via the CD28 pathway augments lymphokine production and proliferation in T cells while preventing induction of anergy [57]. Inactivation of CD28 in vivo gives rise to immune compromised mice, characterized by impaired T-cell responses to antigen and defects in T-cell differentiation [59]. The natural ligands for CD28 are the B7 family of adhesion proteins present on dendritic cells, activated B cells, and macrophages. Ligation of CD28 on T-cells with members of the B7 (CD80 or D86) family on antigen presenting cells provides signal-2. CD28 harbors a YMNM motif in his cytoplasmic tail through which it associates with the p85 subunit of PI3K, a common signaling intermediate, to initiate targeting of AKT (also known as protein kinase B (PKB)) that subsequently results in activation of several distal molecules. Co-stimulatory signals from CD28 ligation results in augmentation of downstream effector cascades, like the PI3 kinase, Ras and acidic sphingomyelinase [57]. These pathways result in the activation of transcription factors such as NF-κB and AP-1, which mediate functional outcomes including IL-2 production and T cell survival [22,45].

Insights for AP-1 involvement in CD28 pathway, comes from in vitro [42,60] and in vivo [61] studies, after CD28 co-stimulation. In part, stimulation of AP-1 activity is a result of JNK activation, which can occur through both TCR/CD3 and CD28 pathways, resulting in higher levels of JNK activity compared with signal 1 stimulation alone [62]. In a study using T-cell blasts, investigators were able to observe the result of CD28 signaling in isolation without the TCR contribution. They found that CD28 costimulation induced AP-1 activity, which was dependent on PI3K and partly the acidic sphingomyelinase [42]. Also c-jun mRNA induction was reported in T-cells after cross-linking of CD28. This CD28-dependent induction of c-jun expression requires protein tyrosine kinase activity but is Ca^2+^ independent [63]. Interestingly, CD28 also recruits the RAS guanine nucleotide exchange factor (GEF) RAS guanyl nucleotide-releasing protein (RASGRP) to the T cell-APC interface to induce activation of RAS and the downstream phosphorylation of AKT, JNKs and ERKs which are potent inducers of the AP-1 activity [64]. Finally, CD28 engagement with B7 ligand, augments JNK signaling, which in turn regulates Elk-1 transactivation at the *c-FOS* gene to promote AP-1 complexes which are important to *IL-2* gene expression [65].

#### 3.1.2. CD40/CD40L

The costimulatory receptor CD40 is a member of the tumor necrosis factor receptor (TNFR) superfamily. CD40 is expressed on dendritic cells, B cells, macrophages and also on non-hematopoietic cells, like endothelial cells and epithelial cells [66]. CD40 binds to its ligand CD40L (or CD154), a type II transmembrane protein, which is transiently expressed primarily on the surface of activated B and T-cells and other non-immune cells [67]. The wide expression pattern of CD40 and its ligand suggests a pivotal role in the regulation of immune-related processes [67]. Ligation of CD40 results in clustering of CD40 and facilitates the recruitment of the TNFR associated factors (TRAFs) to the cytoplasmic domain of CD40 [68]. The TRAFs then activate several signaling pathways including the NFκB, the MAPKs, PI3K, as well as the phospholipase Cγ (PLCγ) pathway [68]. TRAF2 and TRAF3 are involved in activation of the JNK pathway. 

In human urothelial cells, engagement of the CD40 to membrane presented CD40L led to CD40-induced apoptosis involving TRAF3 and JNK/AP-1 activation [69]. Furthermore, the murine *CD40L* promoter contains NFAT binding motifs which require AP-1 cooperational binding for activation of transcription [70]. In biliary epithelial cells, ligation of CD40 with the recombinant CD40L promoted Fas-dependent apoptosis and nuclear factor κB (NF-κB)/AP-1 signaling. Sustained activation of AP-1 in the absence of NF-κB signaling may be critical in determining the outcome of CD40 engagement [71]. Besides, in B-lymphocytes, AP-1 proteins were found to control the mouse IL-6 expression after CD40 engagement, since mutations in the putative AP-1 (and C/EBP) binding sites on the murine *IL-6* promoter, abrogated promoter transcriptional activity. In the same study, CD40 stimulation led to phosphorylation of c-Jun on its activation domain, implicating CD40-mediated Jun kinase activation in the transcriptional regulation of IL-6 production [72]. Another study, also in B cells, has demonstrated that stimulation through both CD40 and Toll-like receptor 7 (TLR7) enhanced the production of cytokines through increased JNK signaling and AP-1 activity. The increased level of active JNK in dual-stimulated cells was accompanied by an increase in the level of active AP-1 monomers cJun and cFos [73]. Moreover, in cultured human fetal microglia cells, ligation of CD40 with soluble trimeric CD40L, results in augmentation of IL-8 (CXCL8) expression and this is mediated by activation of the ERK1/2 MAPK pathway. Gel shift analyses demonstrated that NFκB and AP-1, but not C/EBPβ mediate microglial CXCL8 production [74].

#### 3.1.3. ICOS/4-1BB

In contrast to the constitutively expressed CD28, the inducible T cell co-stimulator (ICOS) has to be de novo induced on the T-cells [75]. ICOS shares several features with CD28 including a YMXM motif in its cytoplasmic tail that associates with p85 of PI3K [76]. ICOS cannot induce *IL-2* gene transcription as efficiently as CD28 and this has been attributed in part to the inability of the YxxM motif of ICOS to associate with Grb2 [38]. ICOS promotes the expansion of several T helper subsets (Th1, Th2, Th17 subsets) and regulatory Treg cells in a context-dependent fashion [57]. AP-1 is involved in ICOS gene expression downstream of TCR/CD28 signaling. An AP-1 binding site was identified on the *ICOS* promoter and previous studies demonstrate that AP-1 binding occurs upon TCR/CD28 stimulation. Moreover, ectopic expression of Fra2 and other AP-1 molecules upregulated ICOS expression on T cells [77].

4-1BB (CD137) is a member of the TNFR superfamily and is responsible for co-stimulation of T-cell responses by interaction with 4-1BB ligand expressed on APC. The expression of 4-1BB has been known to be dependent on T cell activation. Cross-linking of 4-1BB with its ligand, 4-1BBL, promotes IL-2 production, differentiation and proliferation of T-cells while protecting against activation-induced cell death (AICD) of T-cells [78]. Following TCR-stimulation, Kim et al. showed that 4-1BB expression is regulated by the NF-κB and AP-1 transcription factors. By a combination of methods, they identified NF-κB and AP-1 as crucial transcriptional complexes driving 4-1BB transcription and discovered that MEK and JNK1 uncompromised function is required for activation-dependent 4-1BB upregulation [78]. Finally, regulatory AP-1 responsive elements have been found on the promoter of the mouse *4-1BB* gene, suggesting a conserved role for AP-1 among species [79,80].

### 3.2. Co-Inhibitory Molecules

#### 3.2.1. PD-1

Programmed cell death protein-1 (PD-1), also known as CD279, is a type I transmembrane protein, whose expression is induced on activated immune cells such as T, B and NK cells. The major role of PD-1, in contrast to the CTLA-4, is to restrict the T-cell activation in peripheral tissues, to prevent from autoimmune disease and to maintain tolerance within the TME [2]. The cytoplasmic domain of PD-1 contains an immunoreceptor tyrosine-based inhibitory motif (ITIM) and an immunoreceptor tyrosine-based switch motif (ITSM) which are speculated to have immunosuppressive properties [81]. In the direct pathway, binding of PD-1 to its ligand, the B7 member PD-L1, strongly interferes with TCR/CD28 signal transduction and terminates ZAP70 and PI3K phosphorylation by recruiting the SHP1 and SHP2 phosphatases to its tyrosine phosphorylated ITIM and ITSM motifs [82,83]. As a result, PD-1 abrogates cytokine production, causes cell cycle arrest and decreases transcription of the pro-survival factor Bcl-X_L_ [81]. Also, PD-1 inhibits RAS and, subsequently, its downstream targets ERK1 and ERK2 through an SHP1- and SHP2-independent mechanism [57,81]. Because PD-1 is also highly expressed in Tregs where it regulates the development, maintenance, and function of induced regulatory T cells [84], it makes it an ideal target for ICB, since PD-1 inhibition could also interfere with their function on the proliferation on the TME [84].Therefore, PD-1 blockade could theoretically not only lead to enhancement of the activity of effector T cells and NK cells in the peripheral tissues but also to the restriction of the immunosuppressive action of Tregs in the TME [2]. 

A study revealed that a major role of PD-1 is interfering with the AP-1 signaling generated from co-stimulatory cascades. PD-1 inhibits T-cells function by augmenting BATF expression. Accordingly, ectopic expression of BATF was sufficient to impair T cell proliferation and cytokine secretion, whereas BATF knockdown reduced PD-1 inhibition. Silencing BATF in T cells from individuals with chronic viremia rescued HIV-specific T cell function. PD-1, through BATF upregulation, activated a program of genes specific for exhausted CD8^+^ T-cells, although the details of this molecular mechanism still remains elusive [85]. In another study using a mouse model, tumor infiltrating T-cells exhibited high AP-1 activity and specifically expression of c-fos was shown to upregulate PD-1 in tumor infiltrating T-cells during tumor progression. Forced expression of c-fos in T-cells was associated with higher tumor burden, while T-cell specific depletion of c-fos led to reduction in tumor volume. C-fos was found to bind to the promoter region of *PD-1* and thus facilitates its expression. Therefore, blockade of c-fos mediated induction of PD-1 could be harnessed therapeutically to restore T-cells anti-tumor response [86]. 

#### 3.2.2. PD-L1

PD-L1 (CD274), one of the two ligands for PD-1, is a member of the B7 family of co-inhibitory molecules that negatively regulates T-cell immune responses. PD-L1 has a broad expression pattern and it is expressed in normal tissues (T and B cells, NK cells, macrophages, dendritic cells, epithelial cells, and vascular endothelial cells) and tumor cells [87]. Specifically, ligation of PD-L1 of cancer cells to PD-1 expressed on T cells suppresses T-cell activation, proliferation, and induces T-cell apoptosis, which renders it an excellent target for ICB, using antibodies against PD-L1. The regulation of PD-L1 is complex and it occurs at the genetic, transcriptional and post-transcriptional levels [88] and discussing it would be beyond the scope of this review.

In Hodgkin’s Lymphoma (HL), which is characterized by constitutive AP-1 activity [89], AP-1 response elements were identified and demonstrate that cJun and JunB bind to an enhancer region of the *PD-L1* promoter, facilitating the PD-L1 expression along with Epstein-Barr virus (EBV) infection [90]. Also in another EBV-associated tumor, the nasopharyngeal carcinoma (NPC), the EBV-induced latent membrane protein 1 (LMP1) and IFNγ, upregulated PD-L1 expression through AP-1, STAT3 and NF-κB pathways. These findings imply that blocking both the AP-1 oncogenic pathway and PD-1/PD-L1 checkpoints may be a promising therapeutic approach for EBV positive NPC tumors [91].

In melanoma, PD-L1 is highly expressed in cell lines resistant to BRAF inhibitors (BRAFi). BRAFi-resistant cell lines developed dramatic activation in MAPK signaling pathways including extracellular signal-regulated kinase (ERK1/2), JNKs and p38. Increased activation of MAPK promotes PD-L1 expression in the BRAFi-resistant melanoma cells, associated with increased activity of c-Jun. Conversely, inhibition of c-Jun expression by siRNA led to significant decrease of PD-L1 in K028 resistant and parental M34 line, as well as near complete inhibition of PD-L1 expression in M34-resistant line. Thus, c-Jun promotes PD-L1 expression, which can be enhanced via cooperation of STAT3 in melanoma cells. These findings have important therapeutic implications for combining targeted treatment with immune modulation to improve antitumor responses and patient outcomes [92].

In lung adenocarcinoma, MEK inhibition led to a marked reduction on surface PD-L1 levels in vitro, and similar results were seen after ERK2 gene silencing. Moreover, the *PD-L1* promoter was found to contain a functional AP-1 binding site, whose activity is abrogated by MEKi and cJun was bound to this AP-1 site. Overall, the study points to the seminal role of AP-1 in regulating PD-L1 expression, through MAPK upstream signaling [93].

In a mouse model of chronic lymphocytic choriomeningitis virus (LCMV) infection, PD-1 blockade resulted in reinvigoration of exhausted T-cells (T_EX_) but these changes were not accompanied by memory development and T_EX_ become again re-exhausted upon repeated antigen stimulation. The authors hypothesized that the genome-wide epigenetic landscape of T_EX_ may contribute to the lack of durable improvements after PD-L1 checkpoint blockade. Thus, they performed global chromatin landscape mapping using assay for transposase-accessible chromatin with high-throughput sequencing (ATAC-seq). They found that anti-PD-L1 treatment caused transcriptional rewiring and reengagement of effector circuitry in the T_EX_ epigenetic landscape. Motif enrichment analysis of the few differentially accessible regions suggested that cells from anti-PD-L1—treated mice augmented activity of NF-κB, AP-1, and IRF family members but decreased activity of NFAT, Egr2, and Nur77. This study illustrates that AP-1 transcription factors constitute important players in the transcriptional circuitry of re-energized T_EX_ after anti-PD-L1 blockade. [94].

#### 3.2.3. CTLA-4

CTLA-4, a member of the immunoglobulin family, is a CD28 homolog that has higher affinity for B7 ligands [95] and therefore antagonistic actions to CD28. Unlike CD28, which is constitutively expressed on the surface T-cells, CTLA4 is immediately upregulated following TCR engagement (signal-1), with an expression peak, 2 to 3 days after activation [3]. The central role of CTLA4 for keeping T cell activation in check was highlighted from studies with genetically modified mice that are deficient for CTLA-4, which are characterized by profound immune dysregulation and autoimmune disease [96]. It is thus believed that CTLA-4 provides the regulatory braking in proportion to the acceleration received from CD28 and, in contrast to PD-1/PD-L1 axis which function at the peripheral tissues and tumor site, CTLA-4 is regarded as a negative regulator of T-cell function at the site of T-cell priming when naïve T-cells are primed when they are engaged with the peptide-MHC-APC. The inhibitory mechanisms employed by CTLA-4 signals include the recruitment of protein phosphatases, SHP2 and PP2A, which are essential in dampening kinase signals that are induced by TCR/CD28, sequestration of CD80 and CD86 from CD28 engagement as well as active removal of CD80 and CD86 from the APC cell surface [2,97]. Apart from controlling the activation status of CD8^+^effector cells, there is evidence suggesting that CTLA-4 is necessary for the optimal function of Tregs [98] since CTLA-4 is constitutively expressed in Tregs [99]. Treg cell-specific genetic ablation of CTLA4 or blockade with anti-CTLA-4 antibody significantly compromise their ability to regulate both autoimmunity and antitumor immune response [98,100]. Therefore, it is considered that factors contributing to the mechanism of CTLA-4 blockade are the augmentation of both effector CD8^+^ T cell activity and downregulation of Treg cell-dependent immunosuppression [2]. CTLA-4 turns off activation of downstream TCR/CD28 signaling events by inactivation of ERK and JNK pathways, without affecting phosphorylation of TCR-zeta and ZAP70. However, AP-1 activity of these anti-CTLA-4 treated T-cells was not reported in that study [101].

A subsequent study in mouse CD4^+^ T cell blasts illustrated that the suppressive effect exerted by CTLA4 (e.g., on the reduced production of IL-2) is upon inhibiting the signaling delivered through CD28 ligation. Since, CD28 ligation leads to increased transcriptional activity of AP-1 and NF-κB, it was shown that CTLA-4 co-ligation markedly decreased the AP-1 activity on activated T-cells [102]. Findings from another study corroborated the AP-1 role in the immune suppressive function of CTLA-4 ligation, since in CD3/CD28 mAb activated mouse CD4^+^ T-cells, following ligation of CTLA-4, AP-1 and NFAT transcriptional activity was obliterated in a very rapid manner (10 hours after activation). CTLA-4 cross linking on activated cells completely blocked AP-1 and NFAT transcription factors before any effects on T cell proliferation could be observed and importantly they reported that the effect was independent of CD28 co-stimulation, suggesting that CTLA-4 inhibits the TCR signaling cascade [103].

#### 3.2.4. Tregs

Regulatory cells (Tregs) is a population of CD4^+^ T cells that are characterized by the expression of CD25 and the forkhead transcription factor FOXP3 [104,105]. They are also anergic, meaning they don’t produce IL-2 or other effector cytokines such as IFNγ. Lastly, they suppress the immune response of the effector T cells by producing inhibitory factors, such as TGFβ, IL-10 and IL-35. The central role of Tregs is to prevent the excessive activation of immune cells, which would otherwise damage the host but also to suppress anti-tumor immune response. For example, mutations of the gene encoding the Treg-specific transcription factor *FOXP3*, impaired Treg cell development and caused a fatal multi-organ autoimmune disease called immune dysregulation, polyendocrinopathy, enteropathy, and X-linked (IPEX) syndrome [106]. Depletion of CD4^+^CD25^+^FOXP3^+^ Treg cells by a variety of methods is also able to cause similar autoimmune diseases in otherwise normal rodents [107]. Infiltration of a large number of Treg cells into tumor tissues is often associated with poor prognosis. There is accumulating evidence that the removal of Treg cells is able to evoke and enhance anti-tumor immune response [107].

Mantel et al. using a combination of techniques in primary human T-cells, have dissected and functionally analyzed the *FOXP3* promoter. The analysis revealed that the basal promoter contains six NFAT and AP-1 binding sites, which are positively regulating the transactivation of the *FOXP3* promoter after triggering of the TCR [108]. Two regulatory enhancer regions on the *FOXP3* locus, termed the conserved noncoding sequences (CNS) I and II (or Enhancer I and II) were found to control the induction and maintenance of *FOXP3* gene expression. In a more recent study, investigators proposed that AP-1 and other factors control Enhancer 2 activity of the *FOXP3* promoter in Tregs [109].

In naturally occurring Tregs when stimulated with anti-CD3/CD28, FOXP3 was found to maintain the unresponsive state of Tregs by dampening the DNA binding activity of AP-1 transcription factors. Co-immunoprecipitation studies using HEK293 cells indicate that FOXP3 suppresses AP-1 transcriptional activity by interacting with c-Jun. Importantly, JNK-mediated phosphorylation of c-Jun is required for c-Jun and FOXP3 interaction. In addition, FOXP3 also interacts with c-Fos, but not JunB and ATF-2. Finally, inhibition of FOXP3 expression by siRNA in Tregs, restored both AP-1 DNA-binding and the proliferation of Tregs. These data point to a prominent role of FOXP3-AP1-orchestrated regulation of unresponsiveness in Tregs [110].

Another study in a sepsis model of Tregs confirmed that JNK/AP-1 signaling cascade contributes to the elevated expression of FOXP3 and controls *FOXP3* promoter activity. Specifically, c-Jun and c-Fos bind to *FOXP3* promoter, fostering its transcription in Tregs. Accordingly, siRNA treatment of Tregs against AP-1 components, c-Fos, Fra-2, c-Jun or JunD, decreased the expression of FOXP3. Of note, pharmacological inhibition the JNK pathway also reduced FOXPp3 protein levels [111] 

TGF-β is an important contributing growth factor for Treg differentiation and function and has the ability to induce the expression of FOXP3 in CD4^+^CD25^−^ human T cells [112]. In one study investigators used CD4^+^ cells from gene targeted FOXP3-IRES-GFP mice, that were previously stimulated with anti-CD3/CD28^+^ TGF-βand showed that JNK inhibition led to a marked decrease of FOXP3, suggesting that the TGF-β induction of FOXP3 is MAPK-dependent. Moreover, a previously known AP-1 site on the enhancer of *FOXP3* promoter was identified to be a major contributor of the TGF-β-induced *FOXP3* transcriptional activity. In addition, deletion of the AP-1 site in the enhancer of *FOXP3* promoter, greatly decreased *FOXP3* promoter-enhancer activity. Moreover, treatment of anti-CD3/CD28/TGF-β stimulated CD4^+^ T cells with a JNK inhibitor resulted in attenuation of Smad3 binding to enhancer I as determined by ChIP analysis. Taken together, these data indicate that there is control of *FOXP3* transcription exerted by AP-1 either directly (e.g., by direct binding of NFAT/AP-1 complexes on *FOXP3*) or by indirect effects involving the ability of NFAT-AP-1 to regulate the binding of pSmad3 to an adjacent enhancer I site on the *FOXP3* locus [113]. 

The basic leucine zipper transcription factor ATF-like 3 (Batf3) is a member of the AP-1 transcription factor family. Accumulative evidence supports the notion that Batf3 is a key player in Treg differentiation. Specifically, Lee et al. showed that Batf3 is preferentially expressed in effector CD4^+^ T cells and not in Tregs, while it inhibited the differentiation of regulatory T cells in the periphery. Consistently, overexpression of Batf3 in activated naïve CD4^+^ cells inhibited FOXP3 induction. CD4-specific knock out of Batf3 led to favourable differentiation into Tregs in vitro and in colonic lamina propria. Importantly, mice lacking Batf3 showed enhanced Treg function in gut-associated immune disease models. Finally, Batf3 protein was co-precipitated with chromatin from the CNS1 region of the *FOXP3* locus, indicating physical interaction and thereby attenuating gene transcription. Thus, Batf3 is a transcriptional suppressor of Treg differentiation [114]

Another study addressed the role of Batf in Treg biology, by generating strains of mice carrying specific mutant *FOXP3* alleles, that were previously identified in human patients with autoimmune disease driven by globally compromising Treg cell physiology. It was found that a specific *FOXP3* mutation perturbed FOXP3 interactions with Batf by broadening its DNA-binding specificity and induced a distinctive pattern of tissue-restricted inflammation by impairing Treg cell function in certain non-lymphoid tissues. These findings identify Batf as a critical regulator of tissue Treg cell homeostasis [115]

OX40 is a co-stimulatory molecule of the TNFR superfamily, whose expression is rapidly induced by T-cell activation. OX40 costimulation plays a critical role in cell survival, proliferation, and generation of memory cells [116]. Zhang et al. employed naive CD4^+^ T cells activated under iTreg-polarizing conditions with or without OX40 engagement. They showed that OX40 co-stimulation can regulate the differentiation of Tregs, by inhibiting FOXP3 expression and induction of Tregs partly via the AP-1 transcription factor batf3 [117]. They demonstrate that in OX40-stimulated cells, BATF3 and BATF were among a cohort of genes to be highly upregulated. Moreover, upon OX40 ligation in induced Tregs, ChIP assays identified that BATF3/BATF can physically bind to *FOXP3* promoter and through recruitment of Sirt1/7, they produced a closed chromatin configuration at *FOXP3* locus. Thus, targeting OX40 costimulation could have important therapeutic implications [117]. 

A recent study has documented that the AP-1 protein JunB, is expressed in effector Treg (eTreg) cells and is required for eTreg-mediated immune homeostasis through promotion of an interferon regulatory factor 4 (IRF4)-dependent transcription program [118]. In this study investigators showed that mice lacking JunB in Treg cells develop multi-organ autoimmunity, concomitant with aberrant activation of T helper cells. Moreover, they demonstrate that JunB promotes expression of Treg effector molecules, such as ICOS and CTLA4, in BATF-dependent and BATF-independent manners while it is required for homeostasis and suppressive functions of eTreg. Mechanistically, JunB facilitates the accumulation of IRF4 at a subset of IRF4 target sites, including those located near *ICOS* and *CTLA4*. Therefore, this study highlights JunB as a critical regulator of Treg-mediated immune homeostasis [118]. 

## 4. Conclusions

**Figure 2:**
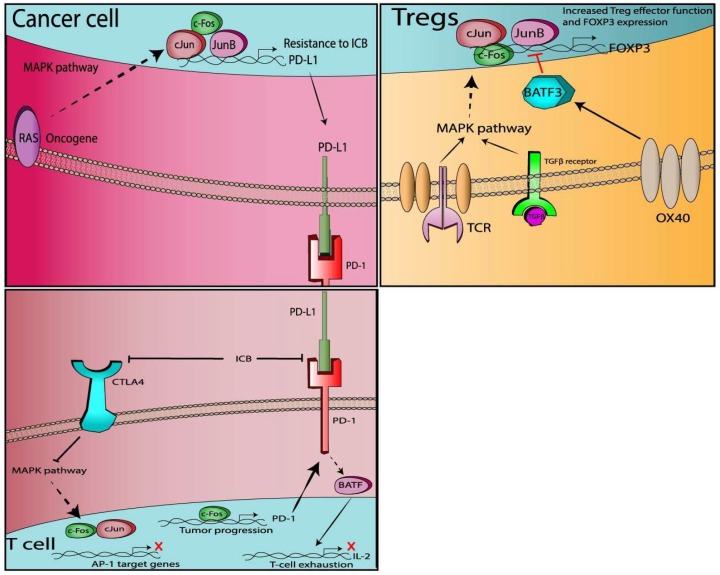
AP-1-dependent regulation of immune checkpoints.

AP-1 components (c-Jun, JunB, c-Fos, Batf) were found to transcriptionally induce the expression of genes encoding for co-inhibitory immune checkpoints (PD-1, PD-L1) via binding on the enhancer regions of the respective gene promoter. On the other hand, studies have demonstrate the ability of AP-1 proteins to bind on *FOXP3* gene locus and promote the expression of this master regulator of Treg identity (Figure 2). Moreover, consistent with a highly context specific and dimerization partner dependent role of AP-1, revisited preclinical data imply differential functions of AP-1 members in Treg physiology, as Batf3 [114] suppresses Treg differentiation while JunB [118] and BatfF [115] appear to positively regulate Treg homeostasis and function. Interestingly, AP-1 complexes seem to participate in the transcriptional circuitry rewiring of exhausted T-cells reinvigoration after ICB, which renders them possible downstream targets of ICB therapy. It is noteworthy that several of these AP-1 related functions responsible for increased inhibitory immune checkpoints expression or Tregs function, were found to be MAPK-dependent [92,93,113], suggesting that they could be attenuated by pathway-specific MAPK inhibitors. Moreover, besides pharmacological inhibition of MAPK, one could speculate that selective depletion of specific AP-1 components (e.g., via siRNA or crisp genetic ablation or novel small molecules) could remove the inhibitory braking exerted by co-inhibitory checkpoints (e.g., PD-1/PD-L1) or impair the immunosuppressive Tregs and thus restore the effective anti-tumor T-cell responses and synergistically augment the efficacy of immunotherapy. As a matter of fact, AP-1 inhibition would be even more advantageous because, in addition to their effects on immune response, they also exert strong tumor growth and survival activity. Unfortunately, AP-1 transcription factors are also crucial in the initial TCR/CD28 downstream cascade which leads to full T-cell activation and is therefore very likely that AP-1 inactivation could undermine the T cell responses that immunotherapy seeks to harness. Much anticipated results from ongoing clinical trials with combinatorial treatments (MAPKi/ICB) might provide some insights on the mechanism of the synergistic effects in cancer patients and whether the AP-1 transcription factors are important mediators. Although more studies are required to elucidate the exact role of this complex family of transcription factors (AP-1) in the regulation of T-cell responses, it is becoming clear that like a delicate balance, AP-1 transcription factors have an impact on both the co-stimulatory and co-inhibitory aspects of T-cell responses (Table 2). Therefore, careful modification of these AP-1 signals could determine the outcome of effector anti-tumor T-cell responses and potentiate the beneficial role of immunotherapy in tumor regression. 

In lung and EBV(+) tumors such as Hodgkin’s lymphoma and nasopharyngeal carcinoma, members of the AP-1 family (cJun and JunB) were found to bind the PD-L1 promoter and induce its expression in a MAPK dependent manner [90,91,92,93]. In a murine model of lung cancer, c-fos overexpression in T-cells, exacerbated tumor progression and led to higher mortality rates compared to control mice. C-Fos forms complexes with cJun and JunB and bind to PD-1 promoter and thus upregulates PD-1 expression in infiltrating T-cells, resulting in attenuated anti-tumor response in this animal model [86]. Moreover, in chronic viral infections, PD-1 expression in T-cells abrogates T-cell proliferation and cytokine expression leading to exhaustion phenotype, through upregulation of the AP-1 protein, BATF [85]. Lastly, studies have demonstrated that CTLA-4 ligation in T-cells disrupts CD28/TCR-dependent signaling and more specifically MAPK (ERK and JNK) activation leading to immune checkpoint inhibition via marked decrease in AP-1 binding activity [101,102,103]. In Tregs, several studies have provided evidence that AP-1 proteins physically bind at regulatory regions of *FOXP3* promoter and facilitate its transactivation [108,109,111]. TCR-TGF-β signaling in Tregs was also shown to induce FOXP3 in a MAPK dependent manner. MAPK downstream cascade leads to the AP-1 activation and transactivation of *FOXP3* [113]. Conversely, induction of the AP-1 transcription factor BATF-3 by OX40 ligation in Tregs, negatively regulates *FOXP3* transcription by physically attaching to the *FOXP3* promoter and attenuated its expression [117]. 

## Figures and Tables

**Table 1:** Ongoing clinical trials with ICB and BRAF (BRAFi) or MEK (MEKi) inhibitors in cancer patients.

National Clinical Trials Identifier	Phase	Targeted Therapy/Immunotherapy	Institution	Scheduling	Indications
NCT02224781	III	D + T, Ipi + Nivo or Ipi + Nivo, D + T	University of Alabama at Birmingham Cancer Center	Sequential	Melanoma
NCT02910700	II	Nivo + T, with or w/o D	M.D Anderson Cancer Center	Concurrent	Melanoma
NCT01940809	I	Ipi w and w/o D, T and/or Nivo	Brigham and Women’s Hospital	Sequential	Melanoma
NCT02130466	I/II	Pembro + T + D	Several locations, USA	Combination	Melanoma
NCT02858921	II	D + T + Pembro	Several location, Australia	Sequential	Melanoma
NCT02625337	II	Pembro + D + T	Antoni van Leeuwenhoek ziekenhuis Amsterdam,	Concurrent	Melanoma
NCT03149029	II	Pembro + D + T	Beth Israel Deaconess Medical Center	Concurrent	Melanoma
NCT02060188	II	Nivo + Ipi + C	Several locations, USA	Concurrent	Colon Cancer
NCT02818023	I	Pembro + V + C	UPMC Cancer Center Hillman Cancer Center	Concurrent	Melanoma
NCT03273153	III	Atezo + C	University of South Alabama; Mitchell Cancer Institute	Concurrent	Melanoma
NCT03013491	I/II	CX-072 (anti-PD-L1) + V	Several locations, USA	Concurrent	Solid tumors and Lymphoma
NCT02968303	II	V + C, Ipi + Nivo	Several locations, The Netherlands	Sequential	Melanoma

Abbreviations: Pembro; pembrolizumab (anti-PD-1), Atezo; atezolizumab (anti-PD-L1), Nivo; Nivolumab (anti-PD-1), Ipi; ipilimumab (anti-CTLA4), CX-072; anti-PD-L1 prodoby, D; dabrafenib (BRAFi), T; trametinib (MEKi), V; vemurafenib (BRAFi), C; Cobimetinib (MEKi).

**Table 2:** Summary of AP-1 interactions in T-cell immune responses.

Gene Name	Direction of the Interaction	AP-1 Member	Mechanism	Activity	Cell Type	References
*CD28*	→ (upregulates)	cJun	PI3K, JNK and ERK dependent	T-cell activation		[42,60,61,62,63]
*ICOS*	← (upregulates)	Fra-2 and other AP-1	TCR/CD28 stimulation/ ICOS promoter binding	Expansion of several T helper subsets and Tregs	T-cells	[77]
*4-1BB*	← (upregulates)	AP-1	4-1BB promoter binding. MEK and JNK dependent.	Co-stimulation of T-cells responses	T-cells	[79,80]
*PD-1*	→ (upregulates)	BATF		Inhibits T-cell function	T-cells in chronic viral infections	[85]
*PD-1*	← (upregulates)	c-Fos	PD-1 promoter binding	Increased tumor burden when expressed in infiltrating T-cells	T-cells in mouse model of lung carcinoma	[86]
*PD-L1*	← (upregulates)	cJun, JunB	PD-L1 promoter binding		EBV(+) tumor cells	[91]
*PD-L1*	← (upregulates)	cJun	MAPK dependent		BRAFi melanoma cell lines	[92]
*PD-L1*	← (upregulates)	cJun	PD-L1 promoter binding. MAPK dependent		Lung carcinoma	[93]
*CTLA-4*	→ (downregulates)	AP-1	MAPK dependent	Inhibits T-cell activation	CD4^+^ T-cells or T-cell blasts	[102,103]
*FOXP3*	→ (downregulates)	cJun, cFos	JNK dependent	Maintains unresponsiveness of Tregs	Natural occurring Tregs	[110]
*FOXP3*	← (upregulates)	cJun, cFos	FOXP3 promoter binding. JNK dependent	Controls FOXP3 promoter activity	Sepsis model of Tregs	[111]
*FOXP3*	← (upregulates)	AP-1	TGFb-induced FOXP3 promoter binding. MAPK dependent	Controls FOXP3 transcriptional activity	Tregs	[113]
*FOXP3*	← (downregulates)	BATF3	FOXP3 promoter binding	Transcriptional suppressor of differentiation of Tregs	Tregs	[114]
*ICOS, CTLA-4*	← (upregulate)	JunB	IRF4 dependent	Loss of JunB in Tregs results in multi-organ autoimmunity	Tregs	[118]
